# Anti-proliferative activity of *A. Oxyphylla* and its bioactive constituent nootkatone in colorectal cancer cells

**DOI:** 10.1186/s12885-020-07379-y

**Published:** 2020-09-14

**Authors:** Eunsu Yoo, Jaehak Lee, Pattawika Lertpatipanpong, Junsun Ryu, Chong-Tai Kim, Eul-Yong Park, Seung Joon Baek

**Affiliations:** 1 Department of Veterinary Medicine, College of Veterinary Medicine and Research Institute for Veterinary Science, Seoul National University, Seoul, 08826 South Korea; 2 Department of Otolaryngology-Head and Neck Surgery, Research Institute and Hospital, National Cancer Center, Goyang, South Korea; 3 R&D Center, EastHill Co. 33, Omokcheon-ro 132 beon-gil, Gwonseon-gu, Suwon-si, Gyeonggi-do 16642 South Korea

**Keywords:** Nootkatone, NAG-1, Cyclin D1, *A. oxyphylla*

## Abstract

**Background:**

*A. oxyphylla* extract is known to possess a wide range of pharmacological activites**.** However, the molecular mechanism of *A. oxyphylla* and its bioactive compound nootkatone in colorectal cancer is unknown.

**Methods:**

Our study aims to examine the role of *A. oxyphylla* and its bioactive compound nootkatone, in tumor suppression using several in vitro assays.

**Results:**

Both *A. oxyphylla* extract and nootkatone exhibited antiproliferative activity in colorectal cancer cells. A. oxyphylla displayed antioxidant activity in colorectal cancer cells, likely mediated via induction of HO-1. Furthermore, expression of pro-apoptotic protein NAG-1 and cell proliferative protein cyclin D1 were increased and decreased respectively in the presence of *A. oxyphylla*. When examined for anticancer activity, nootkatone treatment resulted in the reduction of colony and spheroid formation. Correspondingly, nootkatone also led to increased NAG-1 expression and decreased cyclin D1 expression. The mechanism by which nootkatone suppresses cyclin D1 involves protein level regulation, whereas nootkatone increases NAG-1 expression at the transcriptional level. In addition to having PPARγ binding activity, nootkatone also increases EGR-1 expression which ultimately results in enhanced NAG-1 promoter activity.

**Conclusion:**

In summary, our findings suggest that nootkatone is an anti-tumorigenic compound harboring antiproliferative and pro-apoptotic activity.

## Background

*Alpinia oxyphylla* belongs to the *Zingiberaceae* family and is widely cultivated in Asia as one of the most frequently used plant extracts in oriental medicine. The most well-known medicinal effect of *A. oxyphylla* includes enhancing the internal and astringent activities of the kidney and spleen [1]. Recent studies have shown that *A. oxyphylla* possesses a wide range of pharmacological activities, such as anti-diabetes [2], anti-fibrosis [3], anti-diarrheal [4], and anti-cancer [5].

*A. oxyphylla* contains various chemical constituents, including essential oils, sesquiterpenes, flavones, diarylheptanoids, glycosides and steroids. Amongst them, nootkatone is one of the more abundant components [6]. Nootkatone has also been identified as the main fragrant component of grapefruit with a wide range of beneficial effects including anti-inflammation activities [7], AMPK activation [8] and neuroprotective effects [9]. Other bioactive compounds in *A. oxyphylla,* yakuchinone A [10] and yakuchinone B [11], are also known to have several biological activities including anti-cancer activity. However, the molecular target of nootkatone and other bioactive compounds in cancer or in cell proliferation is unknown.

By analyzing identified molecular targets of phytochemicals, we discovered that the nonsteroidal anti-inflammatory drug [NSAID]-activated gene-1 (NAG-1, also known as GDF15) is highly induced by several phytochemicals [12]. Ectopic expression of NAG-1 causes cell growth arrest, and overexpression of NAG-1 in human colon cells results in reduced tumor formation in the nude mouse model [13]. Although in vitro assays show contradictory results, studies conducted in NAG-1 TG and NAG-1 KO mice consistently demonstrate a clear association between NAG-1 expression and tumor suppression [14]. Thus, NAG-1 induction is a likely molecular mechanism for anticancer activity induced by phytochemicals.

Cyclin D1, another common target of phytochemicals, is often overexpressed in various cancer cell types and tumors. In addition to its role in the cell cycle, cyclin D1 functions as a critical regulator of DNA repair, and thus constitutes a key molecular regulator of transcription [15]. A large number of anticancer chemicals have been shown to downregulate cyclin D1 in various cancer cell types by triggering multiple signaling pathways [16].

EGR-1 is induced very early in the apoptotic process, and mediates the activation of downstream regulators such as p53 [17]. However, EGR-1-induced apoptosis has also been reported in p53^−/−^ cells, indicating the existence of both p53-dependent and p53-independent pathways. EGR-1 also activates tumor suppressor gene phosphatase and tensin homolog (PTEN) during UV irradiation and suppresses the growth of transformed cells both in soft agar and in athymic nude mice [18]. While these results indicate that EGR-1 plays a significant role in growth suppression, the consequences of EGR-1 expression may vary depending on the cellular context. Discrepancies in its role may depend on expression levels of other EGR-1 family members, Sp1, EGR-1 binding repressors, or other factors yet to be identified. Interestingly, EGR-1 has been linked to increased NAG-1 promoter activity, mediated by nonsteroidal anti-inflammatory drugs [19].

Thus, NAG-1, cyclin D1, or EGR-1 could be a molecular target of many bioactive compounds that could lead to anti-proliferation activity. The identification of the molecular target of nootkatone may lead to the development of better single compounds for cancer therapy.

In this study, we identified the biological activity of *A. oxyphylla* and its major compound nootkatone as an inducer of the pro-apoptotic protein NAG-1 and a suppressor of cyclin D1, thereby inhibiting cell proliferation in colon cancer cells. Further, the mechanism by which nootkatone affects cyclin D1 and NAG-1 has been studied. Our results indicated that EGR-1 plays a pivotal role in nootkatone-induced NAG-1 expression, while the proteosomal degradation pathway contributes to nootkatone-mediated cyclin D1 downregulation.

## Methods

### Reagents

*A. oxyphylla* was purchased from Kyung-Dong Market in Seoul, Korea. The authenticity was confirmed at least twice through morphological analysis by Dr. Jaeyoon Cha, Department of Food Science and Nutrition, Dong-A University, Busan, Republic of Korea. A voucher specimen (No. EHNP-H8) has been deposited in the R&D Center, EastHill Corporation, Suwon, Gyeonggi-do, Republic of Korea. The plants were washed and ground using a laboratory mill to a particle size of 100 mesh. Ethanol (70%) was added to the ground plants and extracted at 70 °C for 48 h with stirring at 500 rpm. The extract was filtered using Toyo No. 4 filter paper and concentrated using a vacuum evaporator. Finally, the concentrate was diluted in dimethyl sulfoxide to obtain a final concentration 100 mg/mL. Nootkatone was purchased from Tokyo Chemical Industry (Tokyo, Japan). Epoxomicin and Puromycin (P8833–10) were purchased from Sigma Aldrich (St. Louis, MO, USA) and MG132 was purchased from AdooQ® Bioscience (Irvine, CA, USA). Antibodies for Cyclin D1 (sc-753), HRP conjugated β-actin (sc-47,778), and p53 (sc-126) were purchased from Santa Cruz Biotechnology (Dallas, TX, USA). Antibody for NAG-1 was previously described [13].

### Cell culture

All cells used in this study were purchased from American Type Culture Collection (ATCC). Cells were tested by ATCC for post-freeze viability, growth properties, morphology, mycoplasma contamination, species determination (cytochrome c oxidase I assay and short tandem repeat analysis), sterility test and human pathogenic virus testing. Upon arrival, cell lines were straightaway resuscitated and frozen in aliquots in liquid nitrogen. HCT-116 (Human colorectal carcinoma) and HT-29 (Human colorectal adenocarcinoma) cells were cultured using McCoy’s 5A media (Gibco life technologies, Carlsbad, CA, USA). SW480 (Human colorectal adenocarcinoma), DLD-1 (Human colorectal adenocarcinoma) were cultured using RPMI-1640 media (GIBCO). Both McCoy’s 5A media and RPMI-1640 media contained 10% Fetal bovine serum (FBS; GIBCO) and 1% penicillin/streptomycin (GIBCO). All cells were maintained at 37 °C and 5% CO_2._

### Plasmid transfection and luciferase assay

The promoter-luciferase constructs pNAG1–1086/+ 41, pNAG1–474/+ 41, and pNAG1–133/+ 41 were previously described [12]. The expression vector pcDNA-EGR-1 has also been described [19]. The luciferase and pRL-null plasmids were transfected into cells using PolyJet™ In Vitro DNA Transfection reagent (SignaGen, Frederick, MD, USA) according to manufacturer’s instructions. Luciferase activity was measured using Dual-Luciferase® Reporter Assay kit (Promega, WI, USA) as previously described [20].

### Colony formation assay

Both HCT-116 and SW480 cells were seeded at a density of 1 × 10^4^ cells/well in 6-well culture plates. Cells were treated with nootkatone at various doses (10 μM, 50 μM, or 100 μM) for 9 days. The culture media containing indicated concentrations of nootkatone were changed every 3 days. After treatment, the plate was washed with phosphate-buffered saline, and cells were fixed with 4% paraformaldehyde (Biosesang, Gyeonggi-do, Korea), followed by staining with 1% crystal violet solution (V5265, Sigma Aldrich). The number of colonies was counted using Image J software 1.52a (National Institutes of Health, MD, USA).

### Western blot analysis

Western blot analysis was conducted as previously described [21]. Briefly, 50 μg proteins were separated using 12% sodium dodecyl sulfate-polyacrylamide gel electrophoresis and transferred to a nitrocellulose membrane (GVS filter technology, Zola Predosa BO, Italy). The blotted membrane was then blocked with 5% skim milk for 1 hr at room temperature and incubated overnight with specific antibodies at 4 °C. After incubation with HRP conjugated secondary antibody in 5% skim milk for 1 hr at room temperature, the blotted membranes were visualized using the Alliance Q9 mini imaging system (Cambridge, UK) and quantified using ImageJ software 1.52a (National Institutes of Health, MD, USA).

### RNA isolation and reverse transcription polymerase chain reaction (RT-PCR)

Total RNA was isolated using TRIzol reagent (Invitrogen, Carlsbad, CA, USA). Five hundred nanograms of total RNA was used to synthesize cDNA using Verso cDNA Synthesis kit (Thermo scientific, Waltham, MA, USA). PCR products were amplified using the following primer pairs: cyclin D1 (F: 5′-CAA TGA CCC CGC ACG ATT TC-3′, R: 5′-AAG TTG TTG GGG CTC CTC AG-3′), NAG-1 (F: 5′-CTC CAG ATT CCG AGA CTT GC-3′, R:5′-AGA CAT ACG CAG GTG CAG GT-3′), GAPDH (F: 5′-GAC CAC AGT CCA TGC CAT CAC T-3′, R: TCC ACC ACC CTG TTG CTG TAG-3′). Thermal cycling conditions for NAG-1 were as follows: initial denaturation at 95 °C for 2 min, followed by 25–35 cycles of 94 °C for 30 s, 53.2 °C for 30 s, and 72 °C for 1 min, and final elongation at 72 °C for 5 min. For cyclin D1 and GAPDH, amplification and annealing temperatures were set to 52.5 °C and 60 °C respectively. PCR products were electrophoresed on a 1.5% agarose gel and photographed using the Alliance Q9 mini imaging system.

### Cell proliferation assay

HCT-116 and SW480 were seeded in 96-well plates (1 × 10^3^ cells/well for HCT-116 cells and 2 × 10^3^ cells/well for SW480 cells) and incubated for 24 h with 100 μL of complete medium. Different dose of *A. oxyphylla* was treated for the indicated time. Cell proliferation assays were then performed using CellTiter 96® AQueous One Solution (Promega, WI, USA) according to the manufacturer’s instructions. After indicated time of culture, 20 μL of One Solution reagent was added to each well and cells were incubated for 1 h at 37 °C. Cell viability was estimated by measuring the absorbance at 492 nm using Multiskan FC spectrophotometer (Thermo Fisher Scientific, Waltham, MA).

### Antioxidant activity assay

The 2,2-diphenyl-1-picrylhydrazyl (DPPH, #14805, Cayman Chemical, MI, USA) and 2,2 -azino-bis(3-ethylbenzothiazoline-6-sulfonic acid) (ABTS, #A1888, Sigma Aldrich) were used for the radical scavenging assay, as previously described [22]. The absorbance was measured using a Multiskan™ FC microplate photometer (Thermo Fisher Scientific, Waltham, MA). L-ascorbic acid (#A0537, TCI, Tokyo, Japan) was used as a reference standard in both assays. Determination of the percentage of radical scavenging effect was considered using the following equation:

$$ \%\mathrm{Inhibition}=100-\left[\left(\mathrm{Absorbance}\ \mathrm{of}\ \mathrm{sample}-\mathrm{Absorbance}\ \mathrm{of}\ \mathrm{blank}\right)\times 100/\mathrm{Absorbance}\kern0.5em \mathrm{of}\kern0.5em \mathrm{control}\right]. $$

The VCEAC for ABTS assay and the IC50 value were calculated as half the concentration of the sample that can scavenge 50% of the DPPH free radical.

### Spheroid assay

Seven hundred and fifty HCT-116 cells were seeded in an ultra-low attachment round bottom 96-well plate (Coster, Kennebunk, ME, USA), and cultured for 4 days. After spheroids were formed, half of the media was replaced with complete media containing 2 times the required dosage of nootkatone, and spheroids were incubated for 3 days. Spheroid viability was measured by the CellTiter-Glo® 3D Cell Viability Assay (Promega, Madison, WI, USA) in accordance with the manufacturer’s instuctions. Spheroid volume was calculated using the following formula: 0.5 × Length × Width^2^.

## Statistics

Data are expressed as mean ± SD from at least three independent experiments. Statistical analyses were performed using one-way ANOVA test. All comparisons are relative to untreated or carrier controls and significant differences have been indicated as *p < 0.05; **p < 0.01; ***p < 0.001.

## Results

### Effects of A. oxyphylla extract on cell growth

**Fig. 1:**
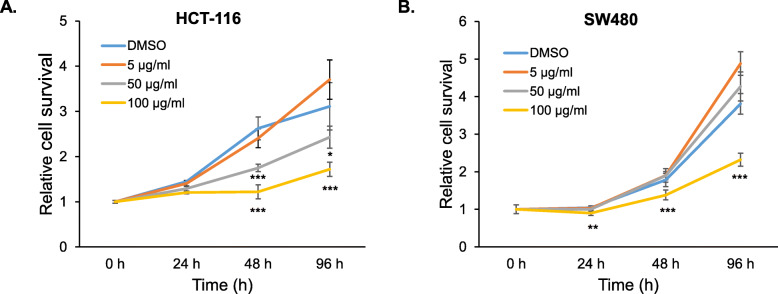
Proliferation assay of colorectal cancer cells in the presence of *A. oxyphylla.* **a** HCT-116 and **b** SW480 cells were treated with various concentrations of *A. oxyphyllal* at different time points*.* Cell numbers were measured by the Cell Proliferation Assay (Promega) after adding an indicated dose of *A. oxyphylla*. DMSO was used as a control. The results from five independent experiments are shown as mean ± SD with statistical significance displayed as *p < 0.05, ***p < 0.001, compared to DMSO-treated cells

*A. oxyphylla* has been used as a traditional Chinese medicine for many years. However, the molecular mechanism of *A. oxyphylla* extract as an anticancer agent has not been elucidated. In the current study, ethanol extracts of *A. oxyphylla* have been obtained and examined for potential antiproliferative activity in two human colorectal cancer cell lines. Treatment with *A. oxyphylla* extract affected HCT-116 and SW480 cell growth in a dose- and time-dependent manner with IC50 values of 89.3 μg/ml and > 100 μg/ml, respectively. (Fig. 1a–b). At 48 h and 96 h, *A. oxyphylla* significantly inhibited cell growth both in p53 wild type (HCT-116) and p53 mutant colorectal cancer cells (SW480), at a concentration of 100 μg/ml.

### Antioxidant activity of *A. oxyphylla* extracts

**Fig. 2:**
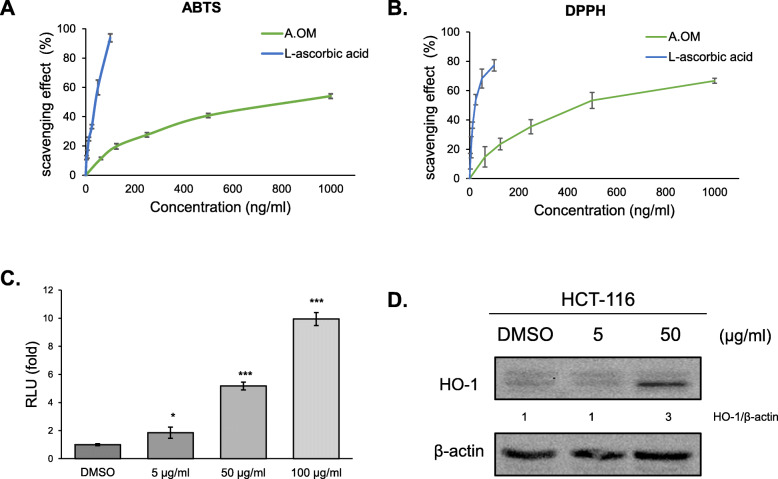
Antioxidant activity of *A. oxyphylla*. **a** 2,2-Azino-bis3-ethylbenthiazoline-6-sulfonic acid (ABTS) radical scavenging ability of the ethanol fraction of *A. oxyphylla*. Vitamin C (L-ascorbic acid) was used as a positive control. Quantification of the result from three independent experiments (*n* = 3) is shown as mean ± SD. **b** The DPPH radical scavenging activities of the ethanol fraction of *A. oxyphyllal*. Quantification of the results from three independent experiments (*n* = 3) is shown as mean ± SD. **c** HCT-116 cells were co-transfected with pARE (Antioxidant response element)-Luc and pRL-null, and luciferase activity was measured. The y-axis shows the number of fold induction of RLU (firefly luciferase activity/Renilla luciferase activity), compared with control of RLU. Quantification of the result from three independent transfections (*n* = 3) is shown as mean ± SD with statistical significance as *p < 0.05, ***p < 0.001. **d** Western blot analysis was conducted to measure HO-1 (Heme oxygenase-1) levels. HCT-116 cell was treated with *A. oxyphylla* extract at various doses for 24 h. β-actin was measured as a loading control for the samples

Antioxidant activity of natural compounds has been shown to be highly related to anticancer effects [23]. Thus, the antioxidant activity of *A. oxyphylla* extract has been investigated. DPPH and ABTS radical scavenging activity assays were performed to determine the antioxidant capacity of *A. oxyphylla* extract at various concentrations. The effects of *A. oxyphylla* extract and ascorbic acid on the ABTS radical compound are shown in Fig. 2a. The ABTS scavenging activities increased in correlation with increasing concentrations of *A. oxyphylla* extract. As a positive control, L-ascorbic acid displayed high antioxidant activity with decreased ABTS radical at a low concentration. Similarly, the DPPH radical scavenging activity of *A. oxyphylla* extract is presented in Fig. 2b. Scavenging activity increased in a dose-dependent manner up to 1000 ng/mL; with a similar trend observed in the ABTS assay. Antioxidant activity was also measured using a luciferase construct containing the antioxidant response element (ARE). After transfection into HCT-116 cells, luciferase activity was increased in the presence of *A. oxyphylla*, suggesting that *A. oxyphylla* may activate NRF2 which binds ARE sites (Fig. 2c). In addition, Heme oxygenase-1 (HO-1) protein levels were analyzed by western blot as a marker for antioxidant activity to confirm the antioxidant effect of the extract. Results showed that HO-1 protein levels increased in a dose-dependent manner (Fig. 2d). Overall, our results indicate that *A. oxyphylla* possesses antioxidant activity.

### NAG-1 and cyclin D1 expression in the presence of *A. oxyphylla*

**Fig. 3:**
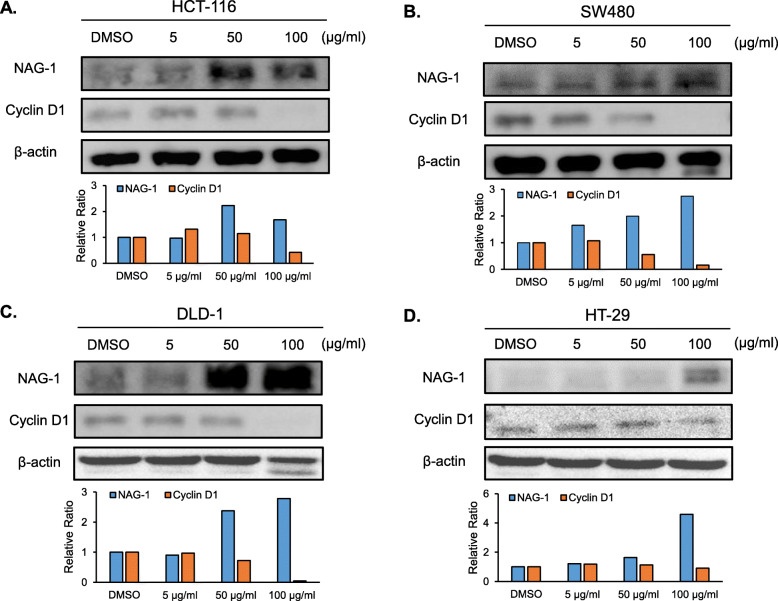
Western blot analysis of NAG-1 and cyclin D1 following *A. oxyphylla* treatment in colorectal cancer cells*.* **a** HCT-116, **b** SW480, **c** DLD-1, and **d** HT-29 cells were treated with *A. oxyphylla* at various concentrations for 24 h in serum-free media. Each cell lysate was subjected to western blot analysis wherein cyclin D1, NAG-1, and β-actin expression were measured. The bar graphs represent the relative protein expression levels of NAG-1 or cyclin D1 after normalization to β-actin

To elucidate the molecular mechanism by which *A. oxyphylla* affects anticancer activity in colorectal cancer cells, expression of NAG-1 and cyclin D1 have been determined. An increase in NAG-1 expression was observed, whereas cyclin D1 expression level was decreased in all four colorectal cancer cell lines treated with *A. oxyphylla* (Fig. 3a–d*)*. These results suggest that *A. oxyphylla* extract may regulate colorectal cancer cell growth by elevating NAG-1 protein expression and decreasing cyclin D1 protein expression.

### Nootkatone exhibits anti-tumorigenic activity in colorectal cancer cells

**Fig. 4:**
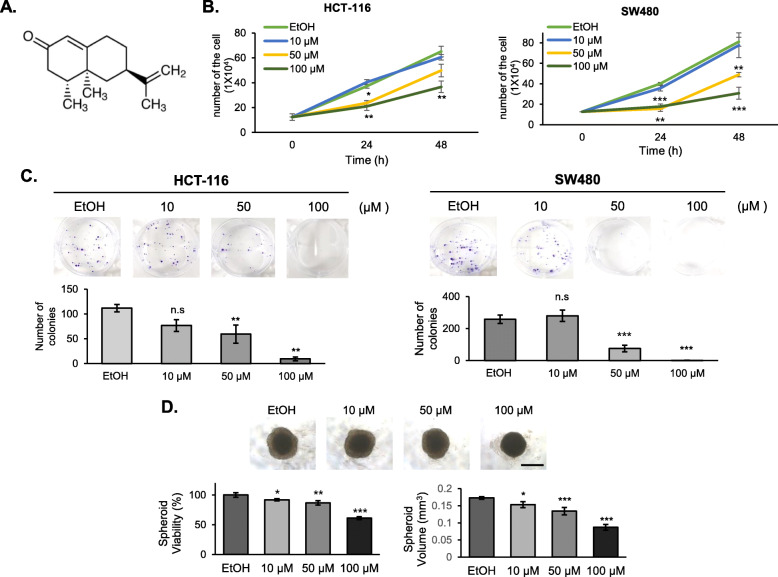
Nootkatone exhibits anticancer activity in colorectal cancer cells. **a** The structure of nootkatone. **b** Cell proliferation assay. HCT-116 and SW480 colorectal cancer cells were treated with various concentrations of nootkatone and cells were counted using hemocytometer. The y-axis shows the cell number and x axis shows the time. Ethanol (EtOH) was used as the vehicle for nootkatone. Quantification of the result from three independent experiments (*n* = 3) is shown as mean ± SD with statistical significance displayed as *p < 0.05, **p < 0.01, and ***p < 0.001. **c** Colony formation assay. HCT-116 and SW480 cells were grown in media containing nootkatone for 9 days. Number of colonies were counted and presented in the bottom graph. The results from three independent experiments (*n* = 3) is shown as mean ± SD with statistical significance displayed as *p < 0.05, **p < 0.01, and ***p < 0.001 compared to EtOH-treated cells. N.S., not significant. **d** Spheroid viability assay. HCT-116 tumor spheroids were treated with nootkatone. Phase-contrast images showed that the size of the spheroid, especially the proliferating zone shrinks in a dose-dependent manner. Scale bars represent 500 μm. *left graph*, spheroid viability was measured by CellTiter-Glo® 3D Cell Viability Assay (Promega). *right graph*, spheroid volume was calculated as described in the Method section. The graph represents three independent experiments. *p < 0.05, **p < 0.01, ***p < 0.001

One of the bioactive compounds found in *A. oxyphylla* is nootkatone. (Fig. 4a) [6]*.* To determine whether nootkatone can account for the antiproliferative effect of *A. oxyphylla*, we analyzed cell growth both by counting cells and by performing colony and spheroid formation assays. Colorectal cancer cells were treated with nootkatone at concentrations of 10 μM, 50 μM and 100 μM, wherein nootkatone treatment resulted in cell growth inhibition in a dose and time dependent manner (Fig. 4b), similar to the trend observed following *A. oxyphylla* extract treatment (Fig. 1)*.* Furthermore, in the colony formation assay, nootkatone showed a dose-dependent inhibition of colony formation in two colorectal cancer cell lines (Fig. 4c). In the spheroid formation assay, nootkatone decreased spheroid formation (viability and volume) in HCT-116 cells (Fig. 4d), indicating that nootkatone possesses anti-tumorigenic activity not only in a 2D culture system, but also in a 3D culture system.

### Nootkatone decreases cyclin D1 and increases NAG-1 expression

**Fig. 5:**
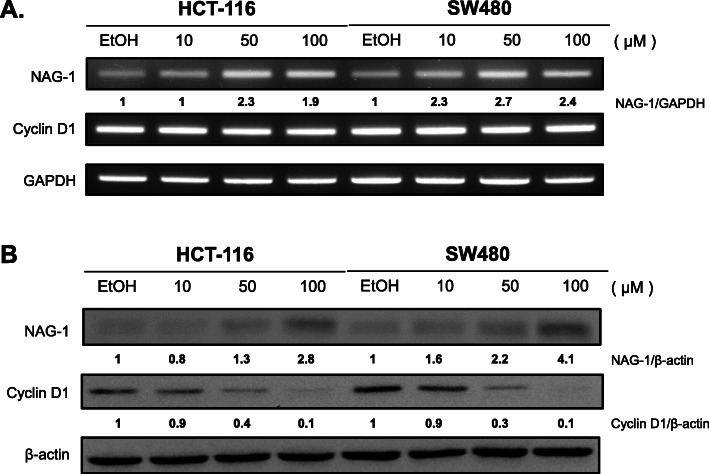
Nootkatone increases NAG-1 and decreases cyclin D1 expression. **a** Total RNA was isolated for RT-PCR analysis from nootkatone-treated HCT-116 and SW480 cells. GAPDH was used as housekeeping control gene. NAG-1 RNA levels increased in a dose-dependent manner, whereas expression level of cyclin D1 did not change. **b** Total proteins were isolated from nootkatone-treated HCT-116 and SW480 cells for western blot analysis. Nooktatone treatment up-regulated NAG-1 protein, while cyclin D1 expression decreased in a dose-dependent manner. β-actin antibody was used as loading control. The relative expression was determined by the Image J program and represented them in the bottom

We examined whether nootkatone may decrease cyclin D1 or increase NAG-1 expression at the transcription level. An increased RNA level of NAG-1 was observed in the presence of nootkatone treatment, whereas cyclin D1 RNA levels did not change in cells treated with nootkatone (Fig. 5a). Protein levels of NAG-1 and cyclin D1 were also analyzed revealing that both NAG-1 and cyclin D1 were altered by nootkatone treatment at the protein level (Fig. 5b). Taken together, our results indicate that nootkatone may affect cyclin D1 at the protein level and NAG-1 at the transcriptional level.

### Nootkatone decreases cyclin D1 expression via proteosomal pathway

**Fig. 6:**
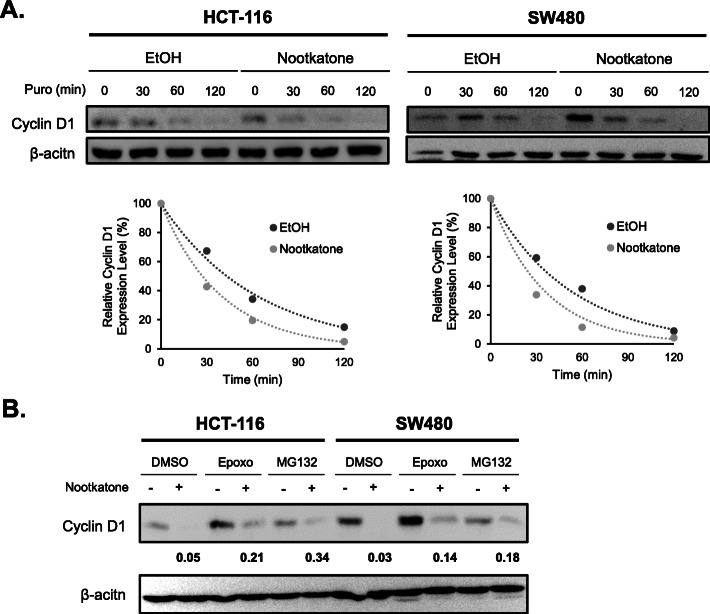
Nootkatone controls cyclin D1 at the protein level. **a** HCT-116 and SW480 cells were pre-treated with 100 μM of nootkatone for 1 h and exposed to 20 μg/ml of puromycin (Puro) at different time points. The cell lysate was harvested at each time point, wherein cyclin D1 and β actin protein levels were detected. Quantitative analysis was performed by Image J. The bottom graph represents degradation of the cyclin D1 protein over time. β-actin was used as loading control. **b** HCT-116 and SW480 cells were pre-treated with DMSO, 10 μM of MG132, or 0.1 μM of epoxomicin, followed by treatment with 50 μM (HCT-116) or 100 μM (SW480) nootkatone for 24 h. β-actin was used as loading control. Quantitative analysis was performed by Image J

To clarify the molecular mechanism by which nootkatone decreases cyclin D1 protein levels, a protein stability assay was performed wherein HCT-116 and SW480 cell lines were treated with puromycin and proteosomal inhibitors. In both cell lines, cyclin D1 protein expression was dramatically decreased by nootkatone treatment compared to the control, indicating that nootkatone may affect the stability of the cyclin D1 protein in the cells (Fig. 6a). Furthermore, proteosomal inhibitors epoxomicin and MG132 were combined with nootkatone to determine whether this would rescue the protein-destabilizing effect of cyclin D1 by nootkatone. Results showed that epoxomicin and MG132 partially restored cyclin D1 protein levels, indicating that nootkatone may affect cyclin D1 degradation through a proteosomal degradation pathway-related mechanism (Fig. 6b).

### Nootkatone increases transcriptional expression of NAG-1 via EGR-1

**Fig. 7:**
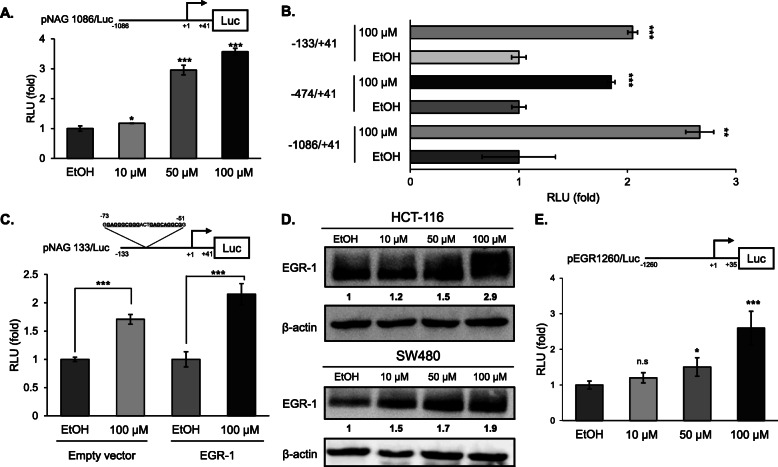
Nootkatone controls the NAG-1 expression at the transcriptional level. **a** Nootkatone increases NAG-1 promoter activity. HCT-116 cells were transfected with pNAG-1 − 1086/+ 41 luciferase and pRL-null plasmid. The cells were treated with EtOH or various concentrations of nootkatone for 24 h, and luciferase activity was measured. The y-axis refers to the ratio of firefly luciferase over renillar luciferase activity. The EtOH-treated cells were set as 1.0. Statistical significance was displayed as *p < 0.05, ***p < 0.001 versus EtOH-treated cells. The data represent mean ± SD from three independent experiments. **b** Three deletion NAG-1 promoter constructs were co-transfected with pRL-null vector into HCT-116 cells. The cells were treated with EtOH or 100 μM of nootkatone for 24 h, and luciferase activity was measured. Fold induction refers to the ratio of luciferase activity in nootkatone-treated cells versus EtOH-treated cells. Statistical significance was displayed as **p < 0.01 and ***p < 0.001 versus EtOH-treated cells. The data represent mean ± SD from three independent experiments. **c** HCT-116 cells were co-transfected with wild type pNAG-1 − 133/+ 41 in the presence of empty or EGR-1 expression vector. Cells were subsequently treated with 100 μM nootkatone for 24 h. The results are presented as means ± S.D. of three independent transfections. **d** Western blot of EGR-1 protein in the presence of nootkatone. β-actin was used as loading control. **e** Luciferase activity of EGR-1 promoter-luciferase construct (pEGR-1260-LUC). The cells were treated with EtOH or nootkatone for 24 h prior to measurement of luciferase activity. Fold induction refers to the ratio of luciferase activity in nootkatone-treated cells compared to EtOH-treated cells. Statistical significance represented as *p < 0.05, ***p < 0.001 versus EtOH-treated cells. n.s. represents not significant. The data represent mean ± SD from four independent experiments

To examine the direct effect of nootkatone on NAG-1 expression, a NAG-1 promoter-luciferase construct containing 1086 bp was transfected into HCT-116 cells and luciferase activity was measured following nootkatone treatment. NAG-1 promoter activity was increased in a dose-dependent manner, with the highest expression corresponding to nootkatone treatment at a concentration of 100 μM (Fig. 7a). To identify the response element region in the NAG-1 promoter which responded to nootkatone, three deletion mutant clone plasmids were designed and analyzed for nootkatone-inducing activity following transfection. The response element position which was the most affected by nootkatone seems to be located within the 133 bp of the NAG-1 promoter (Fig. 6b). It is known that EGR-1 plays a role in transcriptional regulation of NAG-1 in this promoter region [19]. To confirm whether EGR-1 plays a role in nootkatone-induced NAG-1 expression, an EGR-1 luciferase vector was co-transfected with the 133 bp NAG-1 luciferase construct. The results indicated that EGR-1 indeed increased luciferase activity and activated the 133 bp region of the NAG-1 promoter (Fig. 7c). Subsequently, we measured whether EGR-1 protein was increased by the nootkatone treatment. Nootkatone led to increased EGR-1 expression in both HCT-116 and SW480 cells in a dose-dependent manner (Fig. 7d). Finally, we determined whether nootkatone affects EGR-1 expression at the transcriptional level. The result showed EGR-1 promoter activity was increased in the presence of nootkatone in a dose-dependent manner (Fig. 7e), indicating that nootkatone induces EGR-1 at the transcriptional level ultimately leading to the induction of NAG-1 promoter activity.

## Discussion

Plant extracts and its bioactive compounds have been studied in cancer research. Recent data also suggested the usage of plant derived compounds in new generation immunotherapeutic vaccine [24]. *A. oxyphylla* possesses a wide range of biological activities, including anti-diabetes, anti-liver fibrosis, antidiarrheal, and neuronal protection effects [1]. Moreover, several publications have reported anticancer effects of *A. oxyphylla* such as in liver cancer cells via AKT pathway suppression [5]. Our findings further confirm its anti-proliferative activity in colorectal cancer cells. Nootkatone is one of nine bioactive compounds found in *A. oxyphylla* [6], and showed ability to inhibit the expression of inducible nitric oxide synthase (iNOS) and reduce NO production in lipopolysaccharide (LPS) stimulated RAW264.7 cells [25]. Additionally, nootkatone increased survival rates in septic mice by increasing HO-1 expression [25]. Although nootkatone has been linked to anticancer activity in lung cancer via the AMPK pathway [8], the effects of nootkatone against colorectal cancer and its mechanisms underlying these effects remain unknown.

Metastatic cancer encompasses a diverse collection of cells that possess different genetic characteristics and are controlled by many proteins [26–28]. It is also suggested that autophagy plays a role in metastasis [29]. Nootkatone inhibits protein expression that are involved in metastatic cancer and induces autophagy [30]. Thus, molecular elucidation of nootkatone in anti-tumorignesis may lead to better understanding of cancer treatment in metastatic cancer treatment.

In this report, we show that nootkatone treatment contributes to inhibition of cell proliferation in colorectal cancer cells. Our results also suggest that cyclin D1 suppression and NAG-1 induction may at least in part be mechanistically involved in nootkatone-induced anti-tumorigenic activity.

The suppression of cyclin D1 and induction of NAG-1 by nootkatone were observed at a concentration of 100 μM. A similar result was reported in lung cancer cells where 100 μM of nootkatone was required to obtain cell growth inhibition [8]. Since the systemic concentration of nootkatone and its concentration in tissues has not been reported, and most phytochemicals could reach even higher concentrations in the gastrointestinal (GI) track, the speculation that nootkatone probably reaches 100 μM in the GI track is reasonable. Therefore, the large intestinal epithelia, including colon and rectum, could be highlighted as a logical target tissue to further explore nootkatone as an anti-cancer treatment. Additionally, since nootkatone is bio-transformed to various metabolites by fungal strains, the impact of specific nootkatone metabolites may be of particular interest in future cancer studies [31]. In this regard, nootkatone metabolites should be considered as an important compound with respect to cancer progression. A number of reports suggest that cyclin D1 could be a target for many phytochemicals since cyclin D1 downregulation is common in various phytochemical-treated samples [32, 33]. Most phytochemicals affect cyclin D1 at the protein level, as was confirmed in the current study regarding nootkatone’s effects on cyclin D1 protein. Similarly, DIM, EGCG, damnacanthal, and 6-ginerol downregulates cyclin D1 post-translationaly, thereby accounting for the anti-tumorigenic activity of these compounds [16, 32–34]. Cyclin D1 controls many pathways in addition to the cell cycle [35], suggesting that the benefits of cyclin D1 inhibition in cancer may result from several mechanisms.

Transcriptional regulation of NAG-1 is modulated by several *cis*- and *trans*-acting elements [12]. The 133 bp promoter region of NAG-1 contains several transcriptional binding sites, including C/EBPβ [36], p53 [37], EGR-1 [19], and Sp1 [12]. These transcription factors are responsible for the downstream effects of several anticancer compounds including COX inhibitors, PPARγ ligands and cancer chemo-preventive agents [38] which increase NAG-1 transcription. Interestingly, sulindac sulfide (or troglitazone)-mediated NAG-1 up-regulation is dependent on the transcription factor EGR-1 in colon cancer cells [39]. EGR-1 binding sites have been detected in the NAG-1 promoter, overlapping the Sp1 binding site. Here, we report that nootkatone increases EGR-1 both at the protein level as well as at the transcriptional level in colorectal cancer cells, and facilitates NAG-1 promoter activity. This suggests that EGR-1 may be responsible for nootkatone -mediated NAG-1 up-regulation. Since PPARγ ligand troglitazone also increases EGR-1 expression, we examined whether nootkatone may affect PPARγ transcriptional factors. As expected, nootkatone treatment increased PPARγ binding activity as assessed using a reporter construct bearing the PPAR response element (data not shown). Nonetheless, PPARγ activation may not be involved in nootkatone-induced NAG-1 expression at the transcriptional level. Since the emerging pieces of evidence indicate that repurposing of drugs is crucial to the faster and cheaper discovery of anti-cancerous drugs [40], nootkatone should be seriously considered for the design of future cancer drugs.

## Conclusions

Our results indicate that *A. oxyphylla* and its bioactive compound nootkatone exhibit antiproliferative activity in colorectal cancer cells. NAG-1 induction and cyclin D1 downregulation may contribute at least in part to the antiproliferative activity of nootkatone.

## Data Availability

The materials during the current study are available from the corresponding author on reasonable request.

## Abbreviations

NAG-1: Nonsteroidal anti-inflammatory drug activated gene-1
GDF15: Growth differentiation factor 15
EGR-1: Early Growth Response 1
PTEN: Phosphatase and tensin homolog
PPARγ: Peroxisome proliferator-activated receptor gamma
ABTS: 2,2′-Azino-bis(3-ethylbenzothiazoline-6-sulfonic acid)
DPPH: 2,2-diphenyl-1-picrylhydrazyl
C/EBPβ: CCAAT/enhancer-binding protein beta
DIM: 3–3′-di-indolymethane
EGCG: Epigallocatechin gallate
Sp1: *Specificity protein 1*
COX: Cyclooxygenase
HO-1: Heme Oxygenase-1
